# Impact of high myopia on inner retinal layer thickness in type 2 diabetes patients

**DOI:** 10.1038/s41598-023-27529-z

**Published:** 2023-01-06

**Authors:** Jung-Tae Kim, Yong-Jin Na, Sung-Chul Lee, Min-Woo Lee

**Affiliations:** Department of Ophthalmology, Konyang University College of Medicine, #1643 Gwanjeo-dong, Seo-gu, Daejeon, Republic of Korea

**Keywords:** Diseases, Eye diseases

## Abstract

To investigate the impact of the combination of type 2 diabetes (DM) and high myopia on inner retinal layer thickness of the macular area. The patients were divided into four groups: control (group 1), patients with DM without high myopia (group 2), patients with high myopia without DM (group 3), and patients with DM and high myopia (group 4). Ganglion cell complex (GCC) thickness was compared among the groups. Linear regression analysis was performed to identify factors associated with GCC thickness. A total of 194 eyes were enrolled: 59 in group 1, 52 in group 2, 49 in group 3, and 34 in group 4. The average parafovea GCC thicknesses were 113.9 ± 10.4, 112.4 ± 11.2, 112.2 ± 7.8, and 102.6 ± 15.1 μm (P < 0.001), and the average perifovea GCC thicknesses were 104.8 ± 13.2, 103.5 ± 10.8, 103.6 ± 8.8, and 93.9 ± 15.5 μm in groups 1, 2, 3 and 4, respectively (P = 0.001). In multivariate analyses, age (β = − 0.20, P = 0.007), DM duration (β = − 0.34, P = 0.023), and axial length (β = − 1.64, P < 0.001) were significantly associated with parafoveal GCC thickness. The GCC was significantly thinner when high myopia and DM were combined, compared to either condition alone. Additionally, age, DM duration, and axial length were significant factors associated with GCC thickness. The combination of mechanical stretching and neurodegeneration would accelerate neural damage to the retina, resulting in greater inner retinal layer thinning.

## Introduction

Diabetic retinopathy (DR) is the most common complication of diabetes mellitus (DM) and one of the leading causes of blindness among working-age adults in developed countries^1^. Early investigations of DR focused on retinal vascular abnormalities, but emerging studies have shown that retinal thickness and visual function are reduced in patients with DM, even in the absence of clinical DR, which would be associated with diabetic retinal neurodegeneration (DRN). Many studies have reported that DRN predominantly affects the inner retinal layer due to chronic hyperglycemia, oxidative stress, and the accumulation of advanced glycation end products, which can lead to increased glutamate and a loss of neuroprotective factors^2–6^.

High myopia, defined as an axial length ≥ 26.0 mm, is characterized by axial elongation, and consequent stretching of the posterior eye wall can cause retinal detachment, macular holes, choroidal neovascularization, and retinoschisis^7^. Additionally, high myopia is associated with retinal thinning, possibly due to retinal stretching^8^. Many studies have reported that myopia has thinner inner retina than normal individual^9–11^. Mwanza et al. reported a mean decrease in average ganglion cell-inner plexiform layer (GCIPL) thickness of 1.06% per millimeter increase in axial length^12^. Wang et al. also showed negative correlation between GCC thickness and axial length^13^.

Both type 2 DM and high myopia, which are relatively common and increasing in prevalence, greatly accelerate inner retinal layer thinning^14,15^. Lim and colleagues found that simultaneous presence of diabetes and myopia was associated with greater peripapillary retinal nerve fiber layer (RNFL) damage than either pathology alone^16^. However, few studies have assessed inner retinal layer thickness in the macular area in individuals with both DM and high myopia.

The purpose of this study was to investigate the combined impact of DM and high myopia on the inner retinal layer in the macular area, by comparing inner retinal layer thickness between individuals with both conditions and those with only one of them.

## Methods

This retrospective, cross-sectional study adhered to the tenets of the Declaration of Helsinki and was approved by the Institutional Review Board/Ethics Committee of Konyang University Hospital, Daejeon, Korea. We reviewed the charts of patients who visited our retinal clinic from March 2018 to December 2021. The requirement for written informed consent was waived by the Institutional Review Board/Ethics Committee of Konyang University Hospital due to the retrospective nature of the study. We recorded the medical history, best-corrected visual acuity (BCVA), spherical equivalent (SE), intraocular pressure (IOP), and axial length (using an IOLMaster; Carl Zeiss, Jena, Germany, version 5.02) of each patient. The patients were divided into four groups: a control (group 1), patients with DM without high myopia (group 2), patients with high myopia without DM (group 3), and patients with DM and high myopia (group 4). We defined high myopia as an axial length ≥ 26.0 mm. All diabetic patients were initially diagnosed with type 2 DM at the Department of Internal Medicine of Konyang National University Hospital; the diagnosis of DM was made according to the criteria of the American Diabetes Association^17^. The exclusion criteria were any ophthalmic disease that could affect the thickness of the retinal layer, such as glaucoma, retinal disease, or neuro-ophthalmic disease, any history of intraocular surgery except for cataract, an IOP ≥ 21 mmHg, and clinical evidence of DR such as retinal hemorrhages or microaneurysms. We also excluded high myopia patients with structural changes, such as large chorioretinal atrophy, retinoschisis, or posterior staphyloma, which could cause segmentation error when measuring ganglion cell complex (GCC) thickness.

### OCT measurements

Optical coherence tomography (OCT) measurements were performed by a skilled examiner using the Spectralis OCT 2 device (Heidelberg Engineering, Heidelberg, Germany), which can perform 70,000 A-scans per second using a light source centered at 870 nm. A macular volume scan was obtained using spectral domain-OCT (SD-OCT, 25° × 30° field; 25 B-scan lines). Using the Early Treatment Diabetic Retinopathy Study(ETDRS) grid, the macular area was divided into three concentric rings measuring 1, 3, and 6 mm in diameter, centered on the fovea. The two outer rings, with diameters of 3 (defined as the area of parafovea) and 6 mm (defined as the area of perifovea), were divided into four sectors (superior, inferior, nasal, and temporal). Central macular thickness (CMT), defined as the average macular thickness in the central 1-mm area of the ETDRS map, was measured automatically using software bundled with the Spectralis OCT 2 device. Retinal layer segmentation was performed automatically in each horizontal scan. The thickness of the GCC was calculated as the sum of the thicknesses of the macular nerveo fiber layer (NFL), ganglion cell layer (GCL), and inner plexiform layer (IPL) as previous study^18^. We analyzed the data after adjusting for the thickness of each retinal layer for ocular magnification effects based on axial length as in previous studies^19–21^. The actual fundus distance (t) is determined from the OCT measurement (s) by the equation t = p × q × s, where p and q are magnification factors related, respectively, to the imaging system of the OCT device and the eye. Factor q was determined from the Bennett formula: q = 0.01306 × (axial length − 1.82), where 1.82 is a constant related to the distance between the corneal apex and the second principal plane. Factor p for Heidelberg OCT devices is 3.39, given a normal axial length of 24.385 mm.

### Statistical analysis

Demographic characteristics and ocular parameters were compared between groups using one-way analysis of variance followed by a post-hoc test (Bonferroni test), as well as by the Chi-squared test. Univariate and multivariate linear regression analysrs were performed to identify factors associated with the GCC thickness. All statistical analyses were performed with SPSS software (version 18.0; IBM Corp., Armonk, NY, USA).

## Results

### Demographics

A total of 194 eyes were included in this study: 59 in group 1, 52 in group 2, 49 in group 3, and 34 in group 4 (Table 1). The mean age was 52.0 ± 13.6, 54.4 ± 11.2, 52.3 ± 12.5, and 51.5 ± 14.3 years in groups 1, 2, 3, and 4, respectively (P = 0.071). The BCVA of each group was 0.04 ± 0.05, 0.05 ± 0.06, 0.03 ± 0.05, and 0.05 ± 0.07, respectively (P = 0.087). The myopic groups (groups 3 and 4) had a lower SE (P < 0.001) and longer axial length (P < 0.001) than the other groups. Sex, hypertension history, laterality, and CMT were not significantly different among groups. DM duration and HbA1c also showed no significant differences between groups 2 and 4.

**Table 1:** Demographics and clinical characteristics of each group.

	Group 1 (n = 59)	Group 2 (n = 52)	Group 3 (n = 49)	Group 4 (n = 34)	P value
Age (years)	52.0 ± 13.6	54.4 ± 11.2	52.3 ± 12.5	51.5 ± 14.3	0.071
Sex (male, %)	28 (47.5)	34 (65.4)	27 (55.1)	19 (55.9)	0.062
Hypertension (n, %)	19 (32.2)	20 (38.5)	18 (36.7)	11 (32.4)	0.080
DM duration (years)	n/a	7.4 ± 5.9	n/a	8.7 ± 7.3	0.492
HbA1c (%)	n/a	7.5 ± 1.6	n/a	8.3 ± 1.8	0.077
Laterality (right, %)	30 (50.1)	26 (50.0)	30 (61.2)	21 (61.8)	0.747
BCVA (logMAR)	0.04 ± 0.05	0.05 ± 0.06	0.03 ± 0.05	0.05 ± 0.07	0.087
SE (diopter)	− 1.64 ± 2.49	− 0.19 ± 1.88	− 5.97 ± 3.71	− 5.70 ± 4.78	**< 0.001**
IOP (mmHg)	13.9 ± 3.7	13.6 ± 3.6	13.1 ± 3.2	13.6 ± 3.0	0.743
Axial length (mm)	24.4 ± 1.3	23.8 ± 1.1	27.2 ± 1.0	27.4 ± 1.9	**< 0.001**
CMT (μm)	267.5 ± 21.7	271.9 ± 25.2	264.1 ± 19.6	268.8 ± 23.7	0.268

*DM* diabetes mellitus, *BCVA* best-corrected visual acuity, *SE* spherical equivalent, *IOP* intraocular pressure, *CMT* central macular thickness.

All values are expressed as the mean ± standard deviation.

Values in boldface (P < 0.050) are statistically significant.

### Each inner retinal layer and ganglion cell complex thicknesses

The average parafovea NFL thicknesses were 23.9 ± 2.5, 23.2 ± 3.1, 22.9 ± 2.8, and 22.6 ± 3.3 μm (P = 0.048), and the average perifovea NFL thicknesses were 38.2 ± 5.5, 38.3 ± 6.4, 39.7 ± 5.4, and 35.1 ± 8.9 μm (P = 0.012) in groups 1, 2, 3 and 4, respectively. The average parafovea GCL thicknesses were 50.6 ± 5.3, 49.8 ± 6.2, 49.0 ± 5.0, and 43.3 ± 11.0 μm (P < 0.001), and the average perifovea GCL thicknesses were 35.8 ± 3.1, 35.5 ± 3.6, 34.9 ± 3.4, and 31.0 ± 7.0 μm (P < 0.001) in groups 1, 2, 3 and 4, respectively. The average parafovea IPL thicknesses were 41.4 ± 3.7, 40.7 ± 4.0, 40.3 ± 3.2, and 36.6 ± 6.9 μm (P < 0.001), and the average perifovea IPL thicknesses were 31.0 ± 2.4, 29.7 ± 2.6, 29.0 ± 2.2, and 27.8 ± 3.5 μm (P = 0.002) in groups 1, 2, 3 and 4, respectively.

The average parafovea GCC thicknesses were 113.9 ± 10.4, 112.4 ± 11.2, 112.2 ± 7.8, and 102.6 ± 15.1 μm in groups 1, 2, 3 and 4 , respectively (P < 0.001) (Table 2). In post-hoc analyses, group 4 showed a significantly thinner GCC than the other groups (group 1 vs. 4, P < 0.001; group 2 vs. 4, P = 0.001; group 3 vs. 4, P = 0.002). The average perifovea GCC thicknesses were 104.8 ± 13.2, 103.5 ± 10.8, 103.6 ± 8.8, and 93.9 ± 15.5 μm in groups 1, 2, 3 and 4, respectively (P = 0.001). In post-hoc analyses, group 4 showed a significantly thinner GCC than the other groups, similar to the results for the parafoveal area (group 1 vs. 4, P = 0.001; group 2 vs. 4, P = 0.003; group 3 vs. 4, P = 0.003). In analyses of sectoral GCC thicknesses, all sectors except the perifovea superior segment (P = 0.261) showed a significant difference among groups (parafovea superior, P = 0.019; parafovea temporal, P = 0.002; parafovea inferior, P < 0.001; parafovea nasal, P = 0.001; perifovea temporal, P = 0.001; perifovea inferior, P < 0.001; perifovea nasal, P < 0.001).

**Table 2:** Thickness of ganglion cell complex in each group.

	Group 1	Group 2	Group 3	Group 4	P value
**Parafovea**					
Average	113.9 ± 10.4	112.4 ± 11.2	112.2 ± 7.8	102.6 ± 15.1	**< 0.001**
Superior	118.4 ± 13.3	117.5 ± 15.1	116.2 ± 11.3	109.0 ± 18.6	**0.019**
Temporal	106.5 ± 11.1	105.5 ± 11.3	104.4 ± 10.4	95.2 ± 15.2	**0.002**
Inferior	115.1 ± 11.3	113.5 ± 13.4	112.9 ± 13.1	100.1 ± 16.2	**< 0.001**
Nasal	114.8 ± 10.8	112.8 ± 14.6	111.9 ± 10.2	103.2 ± 16.3	**0.001**
**Perifovea**					
Average	104.8 ± 13.2	103.5 ± 10.8	103.6 ± 8.8	93.9 ± 15.6	**0.001**
Superior	107.7 ± 16.3	101.8 ± 14.3	104.5 ± 9.3	96.6 ± 14.9	0.261
Temporal	88.4 ± 7.2	88.5 ± 10.9	88.2 ± 8.9	82.1 ± 12.6	**0.001**
Inferior	105.6 ± 11.0	103.2 ± 11.3	100.9 ± 11.5	89.9 ± 14.5	**< 0.001**
Nasal	119.5 ± 11.7	117.5 ± 15.1	116.7 ± 10.8	106.8 ± 16.7	**< 0.001**

All values are expressed as the mean ± standard deviation (μm).

Values in boldface (P < 0.050) are statistically significant.

### Linear regression analyses of clinical factors and ganglion cell complex thickness

In univariate analyses, age (β = − 0.22, P < 0.001), HTN (β = − 5.00, P = 0.016), DM duration (β = − 0.52, P = 0.001), BCVA (β = − 52.92, P < 0.001), and axial length (β = − 1.12, P = 0.009) were significant factors associated with GCC thickness (Table 3).

**Table 3:** Univariate and multivariate linear regression analyses to identify factors associated with parafoveal ganglion cell complex thickness.

	Univariate		Multivariate	
	B	P value	B	P value
Age	− 0.22 (− 0.34 to 0.10)	**< 0.001**	− 0.20 (− 0.34 to − 0.06)	**0.007**
Sex	− 3.11 (− 6.57 to 0.36)	0.078		
Hypertension	− 5.00 (− 9.08 to − 0.93)	**0.016**	− 1.33 (− 5.55 to 2.89)	0.535
DM duration	− 0.52 (− 0.81 to − 0.23)	**0.001**	− 0.34 (− 0.63 to − 0.05)	**0.023**
Laterality	− 0.84 (− 3.94 to 2.29)	0.599		
BCVA	− 52.92 (− 82.05 to − 23.80)	**< 0.001**	− 19.12 (− 48.58 to 10.34)	0.202
SE	0.34 (− 0.09 to 0.77)	0.120		
IOP	0.36 (− 0.15 to 0.86)	0.169		
Axial length	− 1.12 (− 1.95 to − 0.28)	**0.009**	− 1.64 (− 2.49 to − 0.80)	**< 0.001**

*DM* diabetes mellitus, *BCVA* best-corrected visual acuity, *SE* spherical equivalent, *IOP* intraocular pressure.

Values in boldface (P < 0.050) are statistically significant.

In multivariate analyses, age (β = − 0.20, P = 0.007), DM duration (β = − 0.34, P = 0.023), axial length (β = − 1.64, P < 0.001) were significant.

**Figure 1:**
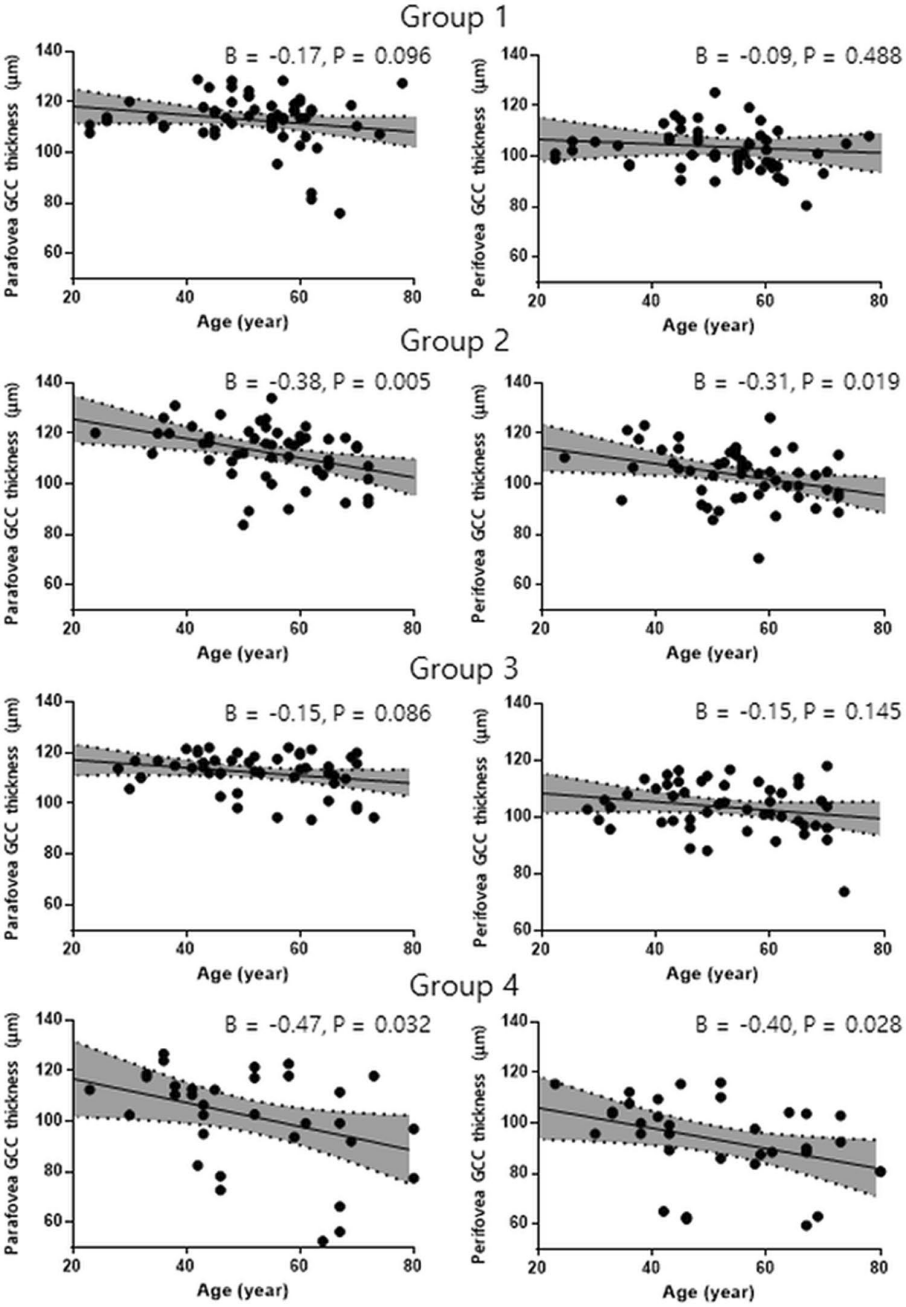
Scatterplots and results of linear regression analyses showing associations between parafoveal ganglion cell complex thickness and age in each group.

**Figure 2:**
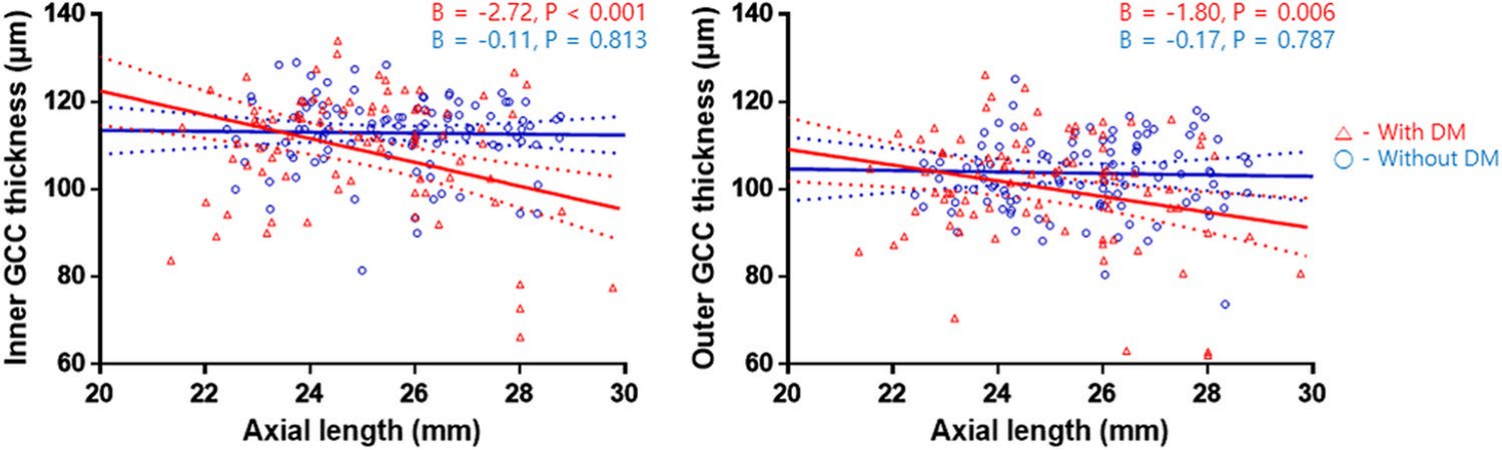
Scatterplots and results of linear regression analyses showing associations between parafoveal ganglion cell complex thickness and axial length in the non-diabetes (DM) groups (groups 1 and 3) and the DM groups (groups 2 and 4).

In group 4, there was a significant negative correlation between GCC thickness and age, with a relatively steep slope (inner: β = − 0.47, P = 0.032; outer: β = − 0.40, P = 0.028) (Fig. 1). Additionally, the axial length was significantly associated with GCC thickness in subjects with DM (inner: β = − 2.72, P < 0.001; outer: β = − 1.80, P = 0.006), but not in those without DM (inner: β = − 0.11, P = 0.813; outer: β = − 0.17, P = 0.787) (Fig. 2).

## Discussion

In this study, we evaluated the GCC thickness of the macular area in control, high myopia, DM, and high myopia and DM groups. The GCC of parafoveal and perifoveal areas was significantly thinner when both diseases were combined. Additionally, age, DM duration, and AL were significant factors associated with GCC thickness in multivariate analyses.

Previous studies have reported inner retinal thinning in patients with DM and high myopia. Lee et al.^22^ reported that the average GCIPL thickness of eyes with high myopia was significantly thinner than those with normal controls (84.29 ± 6.12 vs. 78.50 ± 8.79 μm, P < 0.001). Lim et al.^23^ found that the average GCIPL thickness was 84.23 ± 6.22 and 81.10 ± 4.47 μm in control and DM groups, respectively, which was significantly different (P = 0.001). In our study, groups 2 and 3 showed a tendency for thinner average and sectoral GCC thicknesses than group 1, however, no significant difference was evident in the post-hoc analyses. This discrepancy with previous studies may be due to the use of different OCT devices. The previously mentioned studies used the Cirrus HD OCT system (Carl Zeiss), which measures GCIPL thickness by identifying the outer boundaries of the RNFL and IPL within an annulus with inner vertical and horizontal diameters of 1 and 1.2 mm, and outer vertical and horizontal diameters of 4 and 4.8 mm, respectively. The difference in the area and layer analyzing the inner retina thickness may explain the discrepancies with our findings.

Contrary to the above results, the GCC was significantly thinner in our cases with both DM and high myopia. An earlier study reported that the presence of both DM and myopia was associated with greater peripapillary RNFL damage than that observed with either pathology alone^16^. Accelerated inner retinal thinning by the combination of mechanical stretching in high myopia and DRN would occur not only in the peripapillary area but also in the macular area. Globe elongation may stretch not only retinal tissue but also retinal microvasculature. The stretched microvasculature would be more vulnerable to various damages including pathologic pathways triggered by hyperglycemia compared to normal microvasculature. This can cause severe neurodegeneration by more breakdown of the blood-retinal barrier and neurovascular coupling impairment, which would result in severe inner retinal thinning. Meanwhile, greater peripapillary RNFL damage has been also reported when high myopia and hypertension were both present. Lee et al.^24^ hypothesized that, together, mechanical stretching caused by high myopia and ischemic damage induced by hypertension would lead to a greater reduction in peripapillary RNFL thickness than ischemic or mechanical damage alone. Overall, when high myopia is present together with systemic disease, which damages the inner retina, inner retinal thinning will be accelerated.

Previous studies showed that retinal layer thinning occurs naturally over time due to the effects of aging. Thinning of 0.01–0.16 μm/y of the macular RNFL, 0.05–0.10 μm/y of the GCL, and 0.05 μm/y of the IPL have been described, and tends to be more prominent in older individuals^25–27^. In our study, age was a significant factor associated with GCC thickness, consistent with previous studies. In our subgroup analyses, the GCC thickness of group 2 and 4 patients, who had DM, showed a significant association with age, especially in the latter group. Diffuse loss of neural tissue over time may be accelerated by DRN, especially in patients with high myopia causing mechanical stretching.

Van Dijk et al.^2^ found that the duration of DM was correlated significantly and inversely with GCL thickness; their results suggest that neurodegeneration is primarily caused by a prolonged disturbance of glucose metabolism, which may occur irrespective of the presence of vasculopathy. Lee et al.^28^ also reported that patients with DM duration ≥ 10 years had a thinner GCIPL and lower macular vessel density than those with DM duration < 10 years. Similarly. our study showed a significant association between DM duration and GCC thickness. Once DRN begins, neuronal apoptosis and glial dysfunction may persist and be accumulated over time, resulting in severe inner retina damage in patients with prolonged DM.

Axial length is a known significant factor associated with inner retinal layer thickness. Zhao et al.^9^ reported that the average GCC thickness was significantly associated with axial length. Seo et al.^11^ found a significant correlation between average GCIPL thickness and axial length (− 1.65 μm/mm, P < 0.001). Our multivariate analyses also showed a significant association between axial length and GCC thickness, especilly in patients with DM. This accords with the fact that inner retinal layer damage is exacerbated in the presence of both DRN and mechanical stretching, as mentioned above.

Our study had several limitations. First, it used a retrospective design; thus, the results may not be representative of the general population as selection bias cannot be ruled out. Second, disease duration was based on the date of clinical diagnosis, and thus may have been underestimated. Third, our study was concerned only with the duration of DM, i.e., not the degree of DM control. Despite these limitations, we identified the effects of high myopia and DM, alone and in combination, on inner retinal layer thickness in the macular area, which to our knowledge has not previously been reported.

In conclusion, the GCC was significantly thinner in the presence of both high myopia and DM compared with either condition alone. Additionally, age, DM duration, and axial length were significant factors associated with GCC thickness. In subgroup analyses, the effect of age was greater in patients with both DM and high myopia. Togegher, mechanical stretching and DRN would accelerate neural damage to the retina, resulting in greater inner retinal layer thinning. Physicians should be aware of this when assessing inner retinal layer thickness in patients with DM.

## Data Availability

The datasets used and/or analyzed during the current study available from the corresponding author on reasonable request.
